# What about neurodiversity among nurses? A cross-sectional exploration of work environment and health among nurses with ADHD and/or autism

**DOI:** 10.1177/10519815241289852

**Published:** 2024-12-03

**Authors:** Åsa Hedlund, Malin Jordal

**Affiliations:** Department of Caring Sciences, University of Gävle, Gävle, Sweden

**Keywords:** ADHD, autism, health, nursing, work environment

## Abstract

**Background:**

Employees who have ADHD and/or autism frequently encounter difficulties in their workplace and with their health. Nevertheless, there is a dearth of research focusing on nurses who have ADHD and/or autism, even though the nursing profession presents various significant organizational and social challenges that may potentially have an impact on nurses with ADHD and/or autism.

**Objective:**

The aim of the present study was to describe and examine associations between work environment, perceived health and satisfaction with given care among nurses who have ADHD and/or autism.

**Methods:**

Recruitment of participants took place through Swedish social media groups, and data collection was carried out via a web-based questionnaire. A total of 99 nurses who self-reported a diagnosis of ADHD and/or autism were included in the study. Descriptive statistics, binary correlations and linear regression analyses were conducted to analyze the data.

**Results:**

The nurses reported high quantitative demands at work, low sense of social community and managerial social support, low perceived health and high satisfaction with given care. Regarding associations between variables, nurses with more inattention (ADHD trait) reported higher quantitative demands at work, and nurses with more social anxiety (autistic trait) reported lower perceived health.

**Conclusions:**

Among nurses with ADHD and/or autism, the organizational and social work environment and health are challenging, and these challenges seem to be partly connected to the ADHD and autistic traits. However, patient care was perceived to work well. More research is needed to evaluate how the perceived health of nurses with ADHD/autism can be improved.

## Introduction

Research on neurodiversity, i.e., ADHD (Attention Deficit Hyperactivity Disorder) and autism, in relation to the workplace is an area that has gained increasing attention in recent years, and there is a growing movement advocating for workplaces to be tailored to accommodate ADHD and autism.^1^ Nonetheless, the nursing profession has been somewhat neglected in this area of research. Consequently, there has been a recent increase in scholars advocating for more research in this domain.^2,3^ The present study is a response to this call for action.

ADHD and autism are commonly occurring worldwide. It has been estimated that one percent of the global population has an autism diagnosis^4,5^ and up to 7% an ADHD diagnosis.^6^ Moreover, the numbers are increasing.^4,7^ Notably, there is a significant rate of co-occurrence between these conditions,^8,9^ which means that individuals diagnosed with ADHD often exhibit traits of autism, and vice versa.^10–13^ Common ADHD traits are inattention, hyperactivity and/or impulsivity,^14^ and common autistic traits are difficulties with social interactions and sensations.^15^ The degree of impact on daily life largely depends on the demands and support available within the person's environment.^16–18^ Even though the conditions are currently classified as two distinct diagnoses, they have a great deal in common. For example, they entail similar consequences, such as social challenges and difficulties in managing everyday life.^9^ They are also associated with mental health issues^19,20^ and physical health problems.^21,22^ Therefore, ADHD and autism may be studied together.

It is common for individuals with ADHD and/or autism to experience difficulties in the workplace.^23–26^ For instance, they may experience difficulties in fully utilizing their potential at work, feel that colleagues or supervisors have a negative attitude toward them, and perceive that work performance or job satisfaction is negatively impacted by the work environment not being adapted to ADHD and/or autism.^25,27,28^ However, having ADHD and/or autism does not necessarily have to be experienced negatively by the individual; in fact, it can be a significant part of one's identity and may also bring forth several strengths, such as seeing details easily and thinking creatively.^1,29,30^ Strengths in the workplace related to ADHD and/or autism have been described, such as attention to detail, perseverance, focus, creativity, unique talents, and a strong vested interest in their work.^25,31,32^

The nursing profession is particularly challenging, in that it places significant demands on communication.^33,34^ For instance, nurses are expected to be attentive, express empathy, work effectively in teams, and establish trustful relationships.^33,34^ Furthermore, they need to be able to plan, work in a structured manner, and have a holistic view of the patient.^34^ They also often need to work irregular hours, handle an unpredictable environment, stressful situations, and physical strains such as uncomfortable working positions. All of these aspects may be especially challenging for nurses with ADHD and/or autism. There is generally a lack of research on neurodiversity among healthcare professionals, but some studies have focused on doctors with autism. This research has shown that, according to the doctors themselves, patient care is the aspect that works well, while communication with colleagues and supervisors is less successful. Many doctors expressed a desire for greater understanding of autism in the workplace and for a work environment with fewer sensory stimuli. They also reported experiencing poor mental health.^35^

Thus, it is likely that ADHD and/or autism are of significance in the nursing profession as well, but the specific impact remains unknown. Therefore, the aim of the present study was to describe work environment, perceived health and satisfaction with given care among nurses with ADHD and/or autism. An additional aim was to examine associations between traits related to ADHD and autism and work environment, perceived health and satisfaction with given care.

## Methods

### Design

The present cross-sectional correlational study was conducted following the guidelines outlined in the Strengthening the Reporting of Observational Studies in Epidemiology^36^ checklist, which details essential items to be included in reports of observational studies. A reference group consisting of psychologists, psychiatrists, nurses with ADHD and/or autism as well as researchers in working life and caring science were consulted in the planning of the study to, for example, assess the relevance of design and questionnaires.

### Setting and sampling

The study was conducted with a sample of registered nurses with ADHD and/or autism. To reduce the risk of erroneous results due to insufficient statistical power, we aimed to include at least 85 nurses (significance level 0.05, power 0.8, and effect size 0.35). To determine this, we used a sample size guide from Polit and Beck^37^ for various types of analyses. To be eligible for the study, participants had to be working as nurses and have an ADHD and/or autism diagnosis. They had to report that the diagnosis is confirmed by a doctor. Recruitment occurred through convenience sampling via Swedish Facebook groups dedicated to registered nurses (in general or with ADHD and/or autism). The study invitation, along with the questionnaire link, was shared in these groups after obtaining approval from the group administrator. In total, 100 nurses responded to the invitation. However, one was excluded as he/she had left the nursing profession due to the high workload.

### Data collection

Data were gathered from September to November 2023 using a web-based questionnaire. The questionnaire encompassed areas concerning the nurses’ organizational and social work environment, perceived health, satisfaction with the care provided, as well as items regarding ADHD and autistic traits. The questionnaires are presented below. Additionally, it included background questions such as age, diagnosis, intention to leave, work experience and sick leave.

### Instruments

#### Copenhagen psychosocial questionnaire III (COPSOQ III)

Scales from COPSOQ III were employed to assess organizational and social work environment, i.e., quantitative work demands, social community at work, and managerial social support. COPSOQ III is a widely utilized instrument that has demonstrated satisfactory psychometric properties among Swedish employees.^38^ Quantitative demands at work were assessed using the following items: Is your workload unevenly distributed, leading to a backlog of tasks? How often do you find yourself unable to complete all your assigned tasks? Do you fall behind in your work? Social community was assessed through the following items: Is the atmosphere good between you and your colleagues? Is the cooperation good among colleagues at your workplace? Do you feel involved in a sense of community at your workplace? Social support from the manager was assessed using the following items: If needed, is your immediate manager willing to listen to work-related problems you may have? If needed, do you receive support and assistance with your work from your immediate manager? The response options for all these scales range from ‘Always’ to ‘Never/Almost never’ (100-0), where 100 indicates the highest level of demands, sense of community and social support from the manager. Among Swedish employees in general, the mean value for quantitative demands at work is 40.9, for social community at work is 80.2, and for support from one's manager is 75.3.^38^ In the present study, a mean value ranging from 0 to 100 was computed for each scale and participant, as suggested by the COPSOQ manual.^38^ Internal consistency (Cronbach's alpha) was high, i.e., 0.85 for quantitative demands, 0.80 for social community, and 0.89 for social support from the manager.

#### Perceived health

General perceived health status was assessed using a single item: ‘In general, would you say your health is…’ with response alternatives ranging from poor to excellent (1–4), following the approach outlined by Wang et al.^39^ Single-item subjective health measures are commonly employed due to their simplicity and their effectiveness as indicators of actual morbidity.^40^ Among nurses, higher self-rated health, as measured by a single item, has been found to be correlated with lower levels of burnout.^41^

#### Staff satisfaction with care (SSC)

The nurses’ satisfaction with the care they provided was assessed using a variation of the Staff Satisfaction with Care scale. The original scale was developed specifically for a study by Mårtensson et al.^42^ and has also been slightly modified and utilized in a manner similar to Kaltenbrunner et al.^43^ While it is not a validated instrument, previous studies conducted in a Swedish context have shown that it possesses excellent internal consistency.^42,43^ In the present study, the scale comprises eight items, each offering six response alternatives ranging from ‘Not at all’ (1) to ‘A very high degree’ (6), with 6 indicating the highest level of satisfaction. Sample items include ‘How satisfied are you with the treatments and tests you have conducted for patients?’ and ‘How satisfied are you with the attention you have given to patients’ mental well-being?’ A mean value was computed for each individual for the entire scale. The scale demonstrated excellent internal consistency within the current sample, i.e., 0.91.

#### Adult ADHD self-report scale-6 (ASRS-6)

The ASRS was utilized in the present study to evaluate ADHD traits within the sample. It comprises items aligned with the symptom criteria outlined in the Diagnostic and Statistical Manual of Mental Disorders-5 (DSM-5).^14^ This scale gauges inattention (items 1–4) and hyperactivity/impulsivity (items 5–6) on a 5-point scale (0 = never to 4 = very often), where higher scores indicate greater inattention or hyperactivity/impulsivity. Sample items include: ‘How often do you have difficulty organizing tasks when required?’ (inattention) and ‘How often do you feel excessively active and compelled to engage in activities, as if driven by a motor?’ (hyperactivity/impulsivity). The ASRS-6 has exhibited validity and reliability within a Swedish context.^44^ A total score of 14 or higher (maximum 24) indicates the presence of ADHD.^44^ The sum was calculated for the entire instrument (0–24 points) and for each of its subscales. Internal consistency (Cronbach's alpha) for the present sample was 0.76 for both the total scale and the two subscales.

#### Ritvo autism and asperger diagnostic scale-revised (14 screen) (RAADS-14)

The RAADS-14 assesses autism traits using 14 items distributed across three subscales: Mentalizing deficits (7 items), social anxiety (4 items), and sensory reactivity (3 items). It captures autism symptoms as outlined in the Diagnostic and Statistical Manual of Mental Disorders-5 (DSM-5).^15^ Sample items include: ‘It is challenging to anticipate others’ expectations of me’ (mentalizing deficit), ‘I often struggle with appropriate behavior in social situations’ (social anxiety), and ‘When my senses become overwhelming, I need to isolate myself to cope’ (sensory reactivity).^45^ Response alternatives range from 0 = ‘This has never been true of me and never describes me’ to 3 ‘This is completely true of me now and was when I was younger.’ Higher scores indicate greater mentalizing deficits, social anxiety, and sensory reactivity. The instrument has demonstrated satisfactory validity and reliability within a Swedish context.^45^ A cutoff score of 14 for the entire instrument (range 0–42) has been shown to accurately identify 97% of individuals with autism.^45^ Scores were totaled for the entire instrument and for each subscale. The internal consistency (Cronbach's alpha) was 0.82 for the entire instrument and 0.71 for the mentalizing deficit subscale, 0.66 for social anxiety, and 0.54 for sensory reactivity.

### Data analysis

To analyze the data, IBM SPSS Statistics version 27^46^ was employed. Descriptive statistics were computed for participant characteristics, including age, experience of long-term sick leave due to mental illness, diagnosis, and work experience. The descriptive analyses were conducted on the entire sample and across three diagnostic groups: ADHD only, autism only, and both diagnoses. Subsequently, Spearman's rank correlation coefficient (rho) was utilized to assess linear correlations between the independent variables (ADHD traits: inattention and hyperactivity/impulsivity; autistic traits: mentalizing, sensory sensitivity, and social anxiety) and the dependent variables (quantitative demands, workplace community, support from one's manager, perceived health, and satisfaction with provided care) for the total sample. Variables displaying significant correlations (p < 0.05) with a strength greater than 0.3 (or −0.3)^47^ were intended for use in linear regression analyses to evaluate the impact of independent variables on dependent variables. Long-term sick leave (past and/or present) due to mental illness and self-reported diagnosis were considered potential confounders. Long-term sick leave for mental illness (previous and/or ongoing) was selected as a potential confounder due to its described association with perceptions of the work environment, well-being, and health.^48^ Self-reported diagnosis was selected to ensure that the relationship between the trait(s) and the dependent variable(s) was not solely explained by the diagnosis but was relevant to the sample as a whole. Outliers were absent from the dataset. Missing values accounted for 0.14%, and ‘Exclude cases pairwise’ was employed in SPSS to handle missing data in the analyses.

## Ethics approval and consent to participate

The study was approved by The Swedish Ethical Review Authority in September 2023 (no 2023-03350-01). Written information about the study was provided alongside the questionnaire, and consent to participate was collected from all participants via the initial item in the questionnaire.

## Results

### Participant characteristics

Of the 99 nurses included in the study, a majority (n = 75) reported having an ADHD diagnosis only, followed by individuals with both an ADHD and an autism diagnosis (n = 15). The smallest group consisted of those reporting an autism diagnosis only (n = 9). The nurses ranged in age from 22 to 58 years (mean 36.8), and their nursing experience spanned from 1 month to 37 years (mean 8.2 years). Further participant characteristics can be found in Table 1 and 2.

**Table 1.** Self-reported participant characteristics (n = 99).

Variables	n (%)
**Diagnoses**	
Only ADHD	75 (75.8)
Only autism	9 (9.1)
Both ADHD and autism	15 (15.2)
**Education**	
Only the basic nursing program	64 (64.6)
Specialist nurse^a^	34 (34.4)
Missing	1 (1.0)
**Workload percentage**	
Full-time	53 (53.5)
Part-time	39 (39.4)
Not working at the moment^b^	7 (7.1)
**Long-term sick leave (>2 months) for mental illness during the time as an employed nurse?**	
Yes^c^	52 (52.5)
No	47 (47.5)
**Does the manager know about one's ADHD/autism diagnosis/es?**	
Yes	63 (63.6)
No	33 (33.4)
Not sure	2 (2.0)
Not applicable	1 (2.0)
**Intention to leave within five years**	
No intention to leave	37 (37.4)
Intention to leave one's current workplace	46 (46.4)
Intention to leave the profession	16 (16.2)

^a^For example primary care or childcare.

^b^A majority because of sick leave.

^c^Several of them were on long-term sick leave, part-time or full-time, during the time of data collection.

**Table 2.** Descriptive results for the study variables among the participants (n = 99).

		Mean (SD)			
Variables	Range	Mean total sample (n = 99)	Those with only an ADHD diagnosis (n = 75)	Those with only an autism diagnosis (n = 9)	Those with ADHD and autism diagnosis (n = 15)
**ADHD traits**					
Total score^a^	0–24	15.4 (4.3)	16.2 (3.9)	10.8 (3.6)	14.4 (5.0)
Inattention subscale	0–16	9.7 (3.2)	10.3 (2.9)	6.1 (2.8)	8.7 (3.1)
Hyperactivity/impulsivity subscale	0–8	5.7 (2.0)	5.8 (2.0)	4.7 (1.6)	5.7 (2.1)
**Autism traits**					
Total score^b^	0–42	19.9 (9.9)	17.6 (9.3)	27.3 (7.1)	28.1 (8.0)
Social anxiety	0–12	5.0 (3.6)	4.3 (3.7)	6.4 (2.6)	7.7 (2.2)
Sensory reactivity	0–9	6.1 (2.7)	5.6 (2.7)	8.7 (1.0)	6.8 (2.5)
Mentalizing deficits	0–21	9.1 (5.5)	7.7 (4.8)	13.0 (6.4)	14.1 (4.7)
**Work environment**					
Quantitative demands	0–100	49.3 (22.1)	51.4 (21.4)	47.2 (26.4)	40.0 (22.3)
Social community	0–100	73.4 (17.8)	75.1 (16.0)	75.0 (19.1)	66.1 (24.5)
Managerial support	0–100	63.8 (28.0)	64.2 (27.9)	62.5 (28.0)	62.5 (29.9)
**Perceived health**	1–4	2.2 (0.7)	2.3 (0.7)	2.0 (0.7)	1.9 (0.5)
**Satisfaction with given care**	1–6	4.8 (0.7)	4.8 (0.7)	4.6 (0.8)	4.8 (0.7)

^a^A score >14 indicates that the person has ADHD.

^b^A score >14 indicates that the person has autism.

### Descriptive results

The descriptive findings regarding the study variables revealed, as anticipated, that nurses diagnosed with ADHD exhibited higher scores in both inattention and hyperactivity/impulsivity compared to those diagnosed solely with autism. In turn, nurses with autism demonstrated higher scores in mentalizing deficits, social anxiety, and sensory reactivity in comparison to nurses diagnosed solely with ADHD. However, those with ADHD only (n = 75) scored above the screening threshold for autism, and the total sample (n = 99) scored above the threshold for both ADHD and autism. Regarding work demands, workplace community, managerial support, perceived health, and satisfaction with care provided, the nurses reported relatively high work demands and satisfaction with the care provided, but relatively low levels of workplace community, managerial support, and perceived health. No evident distinctions were seen among the three diagnostic groups. See Table 2 for a detailed description of the various variables for the whole sample and the diagnostic groups.

#### Associations between variables

Correlations between ADHD traits (inattention and hyperactivity/impulsivity) and the dependent variables (work-related quantitative demands, workplace community, managerial support, perceived health, and satisfaction with provided care) revealed that higher inattention among the nurses corresponded to higher perceived quantitative demands at work (rho = 0.33, p < 0.01). Regarding autistic traits (mentalizing, social difficulties, sensory reactivity), the correlations with the dependent variables showed that higher levels of social anxiety among the nurses were associated with poorer perceived health (rho = −0.2, p < 0.05). An overview of the binary correlations can be found in Table 3.

**Table 3.** Correlations (rho) between variables for total sample (n = 99).

	Perceived health	Quantitative demands	Support from manager	Social community at work	Satisfaction with given care
Inattention	0.15	**0** **.** 33**	−0.12	−0.01	0.06
Hyperactivity/impulsivity	−0.07	−0.02	0.15	0.07	0.11
Sensory reactivity	−0.13	−0.12	0.08	−0.04	−0.06
Mentalizing deficits	−0.14	−0.03	−0.10	−0.19	0.01
Social anxiety	−0.20*	−0.04	−0.19	−0.14	−0.09

*Significant at the 0.05 level.

**Significant at the 0.01 level.

The unadjusted linear regression analysis using inattention as the independent variable and quantitative demands as the dependent variable yielded a significant model (p = 0.007) that explained 6.4% of the variance in quantitative demands. It showed that, for every unit increase in inattention, the perception of quantitative demands increased by 1.88 units. Upon inclusion of long-term sick leave due to mental illness (yes/no) and self-reported diagnosis in the model, the overall pattern remained unchanged. Neither sick leave nor self-reported diagnosis exhibited any significant association with quantitative demands. The ADHD trait inattention was still significantly associated with quantitative demands (R2 0.059, B = 1.88). Hence, this single trait could explain perception of quantitative demands at work better than the diagnosis (ADHD/autism) could. An overview of the regression analyses can be found in Table 4.

**Table 4.** Linear regression models with quantitative demands at work^a^ as the outcome (n = 97–98).

Independent variables	B	β	95% CI for B	*p*
*Model 1* ^b^				
Inattention**^c^**	1.88	0.27	0.54–3.23	0.007
*Model 2* ^d^				
Inattention**^c^**	1.88	0.27	0.40–3.35	0.013
Has been on sick leave some time during the time as a nurse^e, f^	−1.31	−0.03	−9.93–7.31	0.764
Self reported diagnosis: Only autism^f, g^	3.50	0.05	−12.82–19.82	0.672
Self reported diagnosis: Both ADHD and autism^f, g^	−8.35	−0.14	−20.66–3.96	0.181

^a^The higher the value, the more quantitative demands.

^b^Adjusted R2 0.064, F(1,97) = 7.72

^c^The higher the score, the more inattention.

^d^Adjusted R2 0.059, F(4,94) = 2.52, p = 0.046

^e^Some were on sick leave during the time of data collection.

^f^Coded as: Yes = 0 (reference category) and No = 1.

^g^Reference category: Only ADHD.

## Discussion

The aim of the present study was to explore work-related quantitative demands, social community at work, managerial support, perceived health, and satisfaction with provided care among nurses who reported having been diagnosed with ADHD and/or autism. Additionally, the study aimed to explore the correlations between traits associated with ADHD and autism and the aforementioned factors. The study revealed a positive significant relationship between inattention (ADHD trait) and perceived quantitative demands at work. This association persisted even after controlling for type of diagnosis.

To be precise, a higher degree of inattention was linked to and explained increased perceived quantitative demands at work. While this finding is notable, it aligns with logical expectations. For instance, it is reasonable to assume that struggles with task organization (i.e., inattention) hinder timely task completion. This is also in line with previous research showing that heightened levels of inattention correlate with increased perceived stress and workplace challenges, such as performance issues and job retention difficulties, among individuals with ADHD.^28,49^ The present study, however, indicates that inattention is a problematic trait in relation to work demands regardless of diagnosis, meaning that support given to prevent the negative consequences of inattention should not be restricted only to those with ADHD.

There is a lack of knowledge regarding how to effectively support individuals experiencing difficulties with inattention within workplace settings,^50^ indicating a need for further research. One complicating factor is that many employees opt not to disclose their diagnosis/diagnoses to their managers. In the present study, more than one-third of the nurses had not divulged their diagnosis to their manager, echoing findings from previous research among doctors with autism.^35^ Qualitative investigations involving workers with ADHD have revealed that this reluctance might stem from fear of adverse repercussions, such as discrimination in the workplace.^31^ Hence, fostering reduced stigma surrounding ADHD and autism within workplaces could prove advantageous for both workers with diagnoses and their managers. A more open environment could facilitate discussions about needs and support.

More than 50% of the nurses had experienced periods of extended absence (>2 months) due to mental illness during their tenure as nurses. Some were still on sick leave at the time of data collection, which may partially account for the relatively low perceived health levels observed in the present study. Considering the common occurrence of psychiatric comorbidities among individuals with autism and ADHD,^19,20^ the elevated prevalence of long-term sick leave among these nurses, although disheartening, might not be surprising. This high prevalence indicates substantial unmet needs within the workplace, further supported by the fact that 62.6% expressed intentions to depart from their current workplace within the next five years. To put this into perspective, Koehler and Olds^51^ conducted a study on the general nurse population (n = 44,082), revealing that only 21% of nurses expressed intentions to leave their current job, even if this was within the timeframe of one year.

Compared to ADHD traits, autism-related traits appeared to pose fewer challenges in the nurses’ professional lives. However, a weak yet significant correlation was observed between a higher level of the autism trait “social anxiety” and lower perceived health. Social anxiety encompasses the ability to navigate group interactions, engage in small talk, navigate social settings, and cultivate friendships.^45^ Nursing work inherently involves numerous social interactions, potentially posing challenges for nurses with autism and/or ADHD and potentially adversely affecting their perceived health. Clarifying mutual expectations in the workplace, something desired by individuals with autism in Tomczak et al.'s study,^52^ could be a strategy for managing social anxiety in the workplace. Quite surprisingly, nurses with ADHD only scored above the screening threshold for autism. It is unclear, however, whether this says something about the sample or whether it says something about the validity of the questionnaire.

Despite the absence of pertinent comparisons in previous literature, it is apparent that satisfaction with provided care remained consistently high across the diagnostic groups. However, concerning managerial support and social community at work, all diagnostic groups exhibited lower levels compared to Swedish employees in general,^38^ but higher quantitative demands than these reference values. Perhaps a more relevant comparison is in relation to other nursing samples in similar contexts. Compared to Swedish hospital nurses in general, nurses in the present study reported higher quantitative demands and lower sense of community at work.^53^ A cautious interpretation of the above-reported results is that organizational and social dynamics within the workplace might be contributing stress factors for nurses with ADHD and/or autism, while their patient care responsibilities could represent a relatively well-functioning aspect of their working life. This is in line with findings from a study involving autistic doctors, where 76% reported encountering challenges in communicating with peers, while only 21% reported similar challenges in communicating with patients.^35^

In summary, few associations were found in this study, which could provide interesting directions for future research. In addition to confirming the results in a larger population, it could also be valuable to validate the scales for work environment and health in individuals with ADHD and/or autism. This is because a different cognitive functioning may lead to different interpretations of the questions. It may also be the case that different questions need to be asked to capture the same phenomenon. For example, research has shown that individuals with autism may have a different understanding from the majority of people regarding what social community consists of.^54^ To gain a more comprehensive understanding of how the work environment functions for these nurses, it would likely be necessary to ask more about stressors that may be particularly challenging for individuals with ADHD and/or autism,^54,55^ such as sensory stimuli in the physical work environment or the structure of the workday.

### Limitations

The present study has several noteworthy limitations, the most significant of which are outlined in this section. Firstly, this was a hard-to-reach group, which resulted in a relatively small sample size selected through convenience sampling via Facebook. This jeopardizes generalizability. The sample was also heterogeneous. While ADHD and autism frequently coexist, they also differ in various respects, and individuals with “autism only” constituted just around 10% of the entire sample. This makes it difficult to generalize the findings specifically to nurses diagnosed solely with autism. Since work experience and age varied so widely, it is also difficult to generalize the results to different age groups and to nurses with varying lengths of experience in the profession. Second, the sample was obtained from social media channels, which may compromise the generalizability of the results. It is plausible that nurses experiencing discomfort may seek peer support on social platforms, thus introducing a bias in the sampled population. Third, some nurses were on full-time sick leave during the data collection. Consequently, their responses to the questionnaires were retrospective, which could have introduced memory bias. Fourth, all data collected were self-reported, undermining the sample's validity. However, considering the elusive nature of this group, recruiting via social media was deemed a reasonably effective method. Notably, participants’ responses aligned as anticipated in the questionnaires. For instance, those with ADHD exhibited higher scores on ADHD-related traits than did those with autism, validating the sample. However, individuals diagnosed solely with ADHD unexpectedly scored higher than anticipated on autism-related traits, surpassing the screening threshold for autism. It is difficult to determine whether this indicates a real overlap between the diagnoses within the sample or whether it is influenced by limitations such as selection bias or lack of questionnaire validity (just because one scores high on a scale, it does not necessarily mean that one actually exhibits those symptoms in real life). Lastly, participant gender was not explicitly queried. However, in the comment section for additional information at the end of the questionnaire (not included in the present study), it was evident that the vast majority of participants were women.

## Conclusions

Nurses diagnosed with ADHD and/or autism experience challenges in their social and organizational work environment, and with their health. Over half of the nurses had experienced long-term sick leave due to mental health issues during their time as employed nurses. Moreover, over half of the nurses intended to leave their work. Nonetheless, the nurses expressed high satisfaction with the care they provided to patients. More research is needed to evaluate how the perceived health of nurses with ADHD/autism can be improved. Based on the present results, it seems to be important for employers and decision makers to target the organizational work environment for these nurses.

## References

[1] Blackburn B . Managing neurodiversity in workplaces. Occup Med (Lond) 2023; 73: 57–58. 36920347 10.1093/occmed/kqac142

[2] Frawley T Gavin B Valeur C , et al. Enhancing the nursing profession’s awareness of neurodiversity. J Clin Nurs 2024; 33: 419–421. DOI: 10.1111/jocn.16902. 38013227

[3] Hedlund Å . Autistic nurses: do they exist? Br J Nurs 2023; 32: 210–214. 36828568 10.12968/bjon.2023.32.4.210

[4] Zeidan J Fombonne E Scorah J , et al. Global prevalence of autism: a systematic review update. Autism Res 2022; 15: 778–790. 35238171 10.1002/aur.2696 PMC9310578

[5] Wang J Ma B Wang J , et al. Global prevalence of autism spectrum disorder and its gastrointestinal symptoms: a systematic review and meta-analysis. Front Psychiatry 2022; 13: 963102. 36081466 10.3389/fpsyt.2022.963102 PMC9445193

[6] Song P Zha M Yang Q , et al. The prevalence of adult attention-deficit hyperactivity disorder: a global systematic review and meta-analysis. J Glob Heal 2021; 11: 04009. 10.7189/jogh.11.04009 PMC7916320 33692893

[7] Abdelnour E Jansen MO Gold J . ADHD Diagnostic trends: increased recognition or overdiagnosis? Mo Med 2022; 119: 467–473. 36337990 PMC9616454

[8] Hours C Recasens C Baleyte J . ASD And ADHD comorbidity: what are we talking about? Front Psychiatry 2022; 13: 837424. 35295773 10.3389/fpsyt.2022.837424 PMC8918663

[9] Rusting R . Decoding the overlap between autism and ADHD. Sci Am 2018.

[10] Leader G Moore R Chen JL , et al. Attention deficit hyperactivity disorder (ADHD) symptoms, comorbid psychopathology, behaviour problems and gastrointestinal symptoms in children and adolescents with autism spectrum disorder. Ir J Psychol Med 2022; 39: 240–250. 33973506 10.1017/ipm.2020.135

[11] Hong JS Singh V Kalb L . Attention deficit hyperactivity disorder symptoms in young children with autism Spectrum disorder. Autism Res 2021; 14: 182–192. 33073542 10.1002/aur.2414

[12] Okyar E Görker I . Examining the autistic traits in children and adolescents diagnosed with attention-deficit hyperactivity disorder and their parents. BMC Psychiatry 2020; 20: 285. 32503560 10.1186/s12888-020-02703-z PMC7275391

[13] Kotte A Joshi G Fried R , et al. Autistic traits in children with and without ADHD. Pediatrics 2013; 132: e612–e622. 10.1542/peds.2012-3947 PMC3876754 23979086

[14] Centers for Disease Control and Prevention. Symptoms and Diagnosis of ADHD. [Internet]. 2023. Available from: https://www.cdc.gov/ncbddd/adhd/diagnosis.html. 2023.

[15] Centers for Disease Control and Prevention. Autism Spectrum Disorder [Internet]. 2022. Available from: https://www.cdc.gov/ncbddd/autism/hcp-dsm.html. 2022.

[16] The Swedish National Board of Health and Welfare. National guidelines for care and support in ADHD and autism. Prioritization support for decision makers and managers. [Internet]. 2022. Available from: https://www.socialstyrelsen.se/globalassets/sharepoint-dokument/artikelkatalog/nationella-riktlinjer/2022-10-8100.pdf. 2022.

[17] World Health Organization. Autism [Internet]. 2023. Available from: https://www.who.int/news-room/fact-sheets/detail/autism-spectrum-disorders?gclid=Cj0KCQiA35urBhDCARIsAOU7QwkTs58CWZ1ZNhdNShdDlsJNo2zrQtGk1Z0I4Yj29qCqRpdo79yBMOgaAsu1EALw_wcB. 2023.

[18] American Psychiatric Association. What is ADHD? [Internet]. 2022. Available from: https://www.psychiatry.org/patients-families/adhd/what-is-adhd. 2022.

[19] Buck TR Viskochil J Farley M , et al. Psychiatric comorbidity and medication use in adults with autism spectrum disorder. J Autism Dev Disord 2014; 44: 3063–3071. 24958436 10.1007/s10803-014-2170-2 PMC4355011

[20] Piñeiro-Dieguez B Balanzá-Martínez V García-García P , et al. CSG. Psychiatric comorbidity at the time of diagnosis in adults with ADHD: the CAT study. J Atten Disord 2016; 20: 1066–1075. 24464326 10.1177/1087054713518240

[21] Pan PY Bölte S . The association between ADHD and physical health: a co-twin control study. Sci Rep 2020; 10: 22388. 33372183 10.1038/s41598-020-78627-1 PMC7769983

[22] Croen LA Zerbo O Qian Y , et al. The health status of adults on the autism spectrum. Autism 2015; 19: 814–823. 25911091 10.1177/1362361315577517

[23] Davies J Heasman B Livesey A , et al. Autistic adults’ views and experiences of requesting and receiving workplace adjustments in the UK. PLoS One 2022; 17: e0272420. 10.1371/journal.pone.0272420 PMC9355205 35930548

[24] Khalifa G Sharif Z Sultan M , et al. Workplace accommodations for adults with autism spectrum disorder: a scoping review. Disabil Rehabil 2020; 42: 1316–1331. 30714420 10.1080/09638288.2018.1527952

[25] Pfeiffer B Braun K Kinnealey M , et al. Environmental factors impacting work satisfaction and performance for adults with autism spectrum disorders. J Vocat Rehabil 2017; 47: 1–12.

[26] Sarkis E . Addressing attention-deficit/hyperactivity disorder in the workplace. Postgr Med 2014; 126: 25–30. 10.3810/pgm.2014.09.2797 25295647

[27] Schiller L . Ny rapport med fokus på arbetsmarknaden och NPF [Internet]. Riksförbundet Attention. 2022. Available from: https://attention.se/2022/11/14/ny-rapport-med-fokus-pa-arbetsmarknaden-och-npf/. 2022.

[28] Fuermaier ABM Tucha L Butzbach M , et al. ADHD At the workplace: ADHD symptoms, diagnostic status, and work-related functioning. J Neural Transm (Vienna) 2021; 128: 1021–1031. 33528652 10.1007/s00702-021-02309-z PMC8295111

[29] Grandin T Panek R . The autistic brain, Thinking across the spectrum. New York: Ebury Press, 2014.

[30] White H Shah P . Creative style and achievement in adults with attention-deficit/hyperactivity disorder. Pers Individ Dif 2011; 50: 673–677.

[31] Oscarsson M Nelson M Rozental A , et al. Stress and work-related mental illness among working adults with ADHD: a qualitative study. BMC Psychiatry 2022; 22: 751. 36451126 10.1186/s12888-022-04409-w PMC9714234

[32] Bury SM Hedley D Uljarević M , et al. The autism advantage at work: a critical and systematic review of current evidence. Res Dev Disabil 2020; 105: 103750. 32810716 10.1016/j.ridd.2020.103750

[33] International Council of Nurses. The global voice of nursing [Internet]. 2023. Available from: https://www.icn.ch/. 2023.

[34] Sjuksköterskeförening S . Kompetensbeskrivning för legitimerad sjuksköterska. [Internet]. 2017. Available from: https://swenurse.se/download/18.9f73344170c003062317be/1584025404390/kompetensbeskrivning legitimerad sjuksköterska 2017.pdf. 2017.

[35] Shaw S Fossi A Carravallah L , et al. The experiences of autistic doctors: a cross-sectional study. Front Psychiatry 2023; 14: 1160994. 37533891 10.3389/fpsyt.2023.1160994 PMC10393275

[36] Checklists – STROBE [Internet]. Available from: https://www.strobe-statement.org/checklists/. 2023 [cited 2021 Aug 30].

[37] Polit D Beck C . Nursing research. Generating and Assessing Evidence for Nursing Practice. 11^th^ ed. Philadelphia: Wolters Kluwer Health, 2020.

[38] Berthelsen H Westerlund H Bergström G , et al. Validation of the Copenhagen psychosocial questionnaire version III and establishment of benchmarks for psychosocial risk management in Sweden. Int J Env Res Public Heal 2020; 17: 31–79. 10.3390/ijerph17093179 PMC7246423 32370228

[39] Wang MT Muntz A Wolffsohn JS , et al. Association between dry eye disease, self-perceived health status, and self-reported psychological stress burden. Clin Exp Optom 2021; 104: 835–840. 33689664 10.1080/08164622.2021.1887580

[40] Palladino R Tayu Lee J Ashworth M , et al. Associations between multimorbidity, healthcare utilisation and health status: evidence from 16 European countries. Age Ageing 2016; 45: 431–435. 27013499 10.1093/ageing/afw044 PMC4846796

[41] Malagón-Aguilera MC Suñer-Soler R Bonmatí-Tomas A , et al. Dispositional optimism, burnout and their relationship with self-reported health Status among nurses working in long-term healthcare centers. Int J Env Res Public Heal 2020; 17: 4918. 10.3390/ijerph17144918 PMC7399977 32650418

[42] Mårtensson G Carlsson M Lampic C . Is nurse-patient agreement of importance to cancer nurses’ satisfaction with care? J Adv Nurs 2010; 66: 573–582. 20423392 10.1111/j.1365-2648.2009.05228.x

[43] Kaltenbrunner M Bengtsson L Mathiassen SE , et al. Staff perception of lean, care-giving, thriving and exhaustion: a longitudinal study in primary care. BMC Heal Serv Res 2019; 19: 652. 10.1186/s12913-019-4502-6 PMC6734292 31500624

[44] Lundin A Kosidou K Dalman C . Testing the discriminant and convergent validity of the world health organization six-item adult ADHD self-report scale screener using the Stockholm public health cohort. J Atten Disord 2019; 23: 1170–1177. 29073818 10.1177/1087054717735381

[45] Eriksson JM Andersen LM Bejerot S . RAADS-14 Screen: validity of a screening tool for autism spectrum disorder in an adult psychiatric population. Mol Autism 2013; 4: 49. 24321513 10.1186/2040-2392-4-49 PMC3907126

[46] IBM Corp. IBM SPSS Statistics for Windows, Version 27.0. Armonk, NY: IBM Corp, 2020.

[47] Pallant J . SPSS Survival Manual. 6th ed. Milton Keynes, United Kingdom: Open University Press, 2016.

[48] Joosen MCW Lugtenberg M Arends I , et al. Barriers and facilitators for return to work from the perspective of workers with common mental disorders with short, Medium and long-term sickness absence: a longitudinal qualitative study. J Occup Rehabil 2022; 32: 272–283. 34580811 10.1007/s10926-021-10004-9 PMC9232415

[49] Combs MA Canu WH Broman-Fulks JJ , et al. Perceived stress and ADHD symptoms in adults. J Atten Disord 2015; 19: 425–434. 23034340 10.1177/1087054712459558

[50] Lauder K McDowall A Tenenbaum H . A systematic review of interventions to support adults with ADHD at work-implications from the paucity of context-specific research for theory and practice. Front Psychol 2022; 13: 893469. 36072032 10.3389/fpsyg.2022.893469 PMC9443814

[51] Koehler T Olds D . Generational differences in Nurses’ intention to leave. West J Nurs Res 2022; 44: 446–455. 33745402 10.1177/0193945921999608

[52] Tomczak MT Mpofu E Hutson N . Remote work support needs of employees with autism Spectrum disorder in Poland: perspectives of individuals with autism and their coworkers. Int J Env Res Public Heal 2022; 19: 10982. 10.3390/ijerph191710982 PMC9518488 36078696

[53] Ulhassan W von Thiele Schwarz U Thor J , et al. Interactions between lean management and the psychosocial work environment in a hospital setting - a multi-method study. BMC Heal Serv Res 2014; 14: 480. DOI: 10.1186/1472-6963-14-480 PMC4282497 25339236

[54] Schreuer N Dorot R . Experiences of employed women with attention deficit hyperactive disorder: a phenomenological study. Work 2017; 56: 429–441. 28269805 10.3233/WOR-172509

[55] Diener ML Wright CA Taylor C , et al. Dual perspectives in autism spectrum disorders and employment: toward a better fit in the workplace. Work 2020; 67: 223–237. 32955484 10.3233/WOR-203268

